# Sumoylation and the DNA Damage Response

**DOI:** 10.3390/biom2030376

**Published:** 2012-09-04

**Authors:** Catherine A. Cremona, Prabha Sarangi, Xiaolan Zhao

**Affiliations:** 1 Molecular Biology Program, Memorial Sloan-Kettering Cancer Center, New York, NY 10065, USA; Email: catherine.cremona@cancer.org.uk (C.A.C.); prs2007@med.cornell.edu (P.S.); 2 Programs in Biochemistry, Cell, and Molecular Biology, Weill Cornell Graduate School of Medical Sciences, New York, NY 10065, USA

**Keywords:** sumoylation, DNA damage response, genome integrity

## Abstract

The cellular response to DNA damage involves multiple pathways that work together to promote survival in the face of increased genotoxic lesions. Proteins in these pathways are often posttranslationally modified, either by small groups such as phosphate, or by protein modifiers such as ubiquitin or SUMO. The recent discovery of many more SUMO substrates that are modified at higher levels in damage conditions adds weight to the accumulated evidence suggesting that sumoylation plays an important functional role in the DNA damage response. Here we discuss the significance of DNA damage-induced sumoylation, the effects of sumoylation on repair proteins, sumoylation dynamics, and crosstalk with other posttranslational modifications in the DNA damage response.

## 1. Introduction

**Figure 1:**
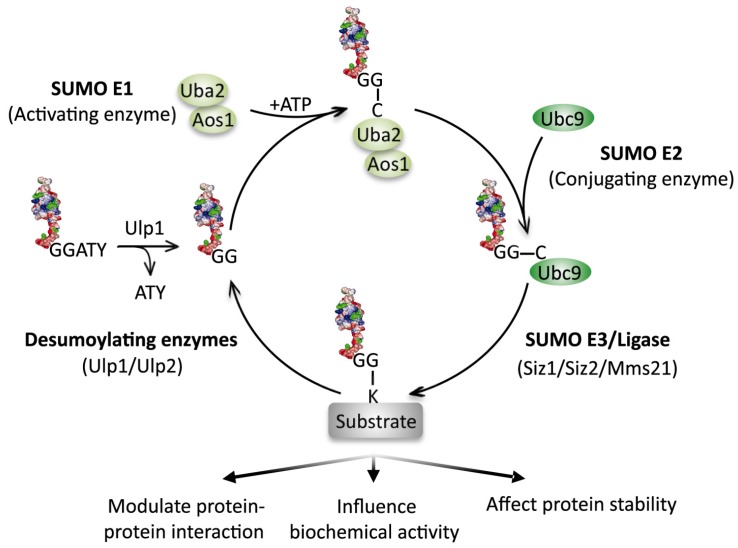
The sumoylation cycle and three commonly seen effects of substrate sumoylation. For simplicity, only enzymes from *S. cerevisiae* are shown. SUMO is processed by the Ulp1 protease to expose a diglycine (GG) motif, which is adenylated and transferred to cysteine (C) residues in SUMO E1 and then E2 enzymes. SUMO is conjugated to a lysine residue (K) or residues (not shown) on the substrate either directly by the E2, or more frequently with the help of SUMO E3s. The SUMO moiety is shown as the surface representation of the SUMO-1 structure [18,19]. Poly-SUMO chains can form when SUMO is conjugated to lysines on the protein itself (not shown).

Maintenance of genome integrity is a dynamic task, requiring a fast and coordinated response to increased DNA damage from either exogenous genotoxins or endogenous lesions. This is known as the DNA damage response (DDR), and entails an elaborate signaling network that connects the detection of damage to chromatin modifications, cell-cycle control, and various modes of DNA repair, as well as other adaptive changes in cell physiology [1]. Sumoylation, the covalent linkage of a small protein, SUMO (approx. 100 amino acids), to a lysine residue or residues on substrate proteins, has been implicated in this response. The enzymatic pathways of sumoylation and desumoylation have been reviewed elsewhere [2,3,4] and are briefly summarized in Figure 1. SUMO is first processed from an inactive precursor by a SUMO protease that removes a few C-terminal amino acids. This conjugation-proficient form then undergoes three enzymatic steps, catalyzed sequentially by SUMO E1, E2, and E3 enzymes, to become covalently linked to a lysine residue on a substrate. Substrates can be poly-sumoylated on one residue or multiply sumoylated on several residues. The removal of SUMO is catalyzed by several desumoylation enzymes. Like other posttranslational modifications, SUMO enables rapid and reversible modulation of protein function via changes in enzymatic activity, conformation, interactions, or stability (Figure 1). At the molecular level, such changes allow proteins to achieve the complex choreography necessary to carry out their roles. At the cellular level, this molecular flexibility permits fast adaptation to changing conditions, promoting survival. Sumoylation, like other posttranslational modifications, is therefore well suited to managing and fine-tuning the DDR network. Several recent reviews have described various contributions of sumoylation to genome integrity, both alone and in collaboration with other modifications [5,6,7,8,9,10,11,12,13]. In addition, specific functions of sumoylation in DNA repair, such as stimulating BRCA1’s ubiquitin ligase activity, helping to recruit repair proteins to double-strand breaks [14,15], and facilitating the release of the homologous recombination protein Rad52 from DNA [16], have been reviewed in the last issue and an extensive list of SUMO substrates involved in DNA replication and repair has been compiled [17].

Most relevant to this review, the list of sumoylation substrates modified in response to damage has recently expanded [20,21]. This indication that sumoylation may play a wider role in the DNA damage response than was previously appreciated raises several questions. What is the precise function of sumoylation in each of these cases? It is already clear that there is no single outcome of sumoylation for its target proteins, but common themes may be shared amongst certain substrates. How is sumoylation controlled—via activation of a central DDR enzyme, like the ATM/ATR DNA damage checkpoint, or via regulation of the substrate? What is the relative contribution of desumoylation to overall sumoylation levels in stress-induced and normal conditions, and to protein function? How do sumoylation events integrate with other modifications at damage sites? This review will discuss possible answers to these questions, with reference to our own work in budding yeast as well as recent research from other laboratories in yeast and mammalian systems.

## 2. The Significance of DNA Damage-Induced Sumoylation (DDIS)

The observation that sumoylation of many replication and repair proteins increases following DNA damage immediately offers the attractive theory that this response helps to facilitate replication and bring about repair. The recruitment of PIAS SUMO E3s (homologs of yeast Siz1 and Siz2) to double-strand break (DSB) sites in human cells and the impairment of homologous recombination (HR) when sumoylation is defective support this idea and provide strong evidence that sumoylation is playing a functional role in the damage response to DSBs [14,15,20,22,23,24,25,26]. Our results further showed that one of the roles of sumoylation in the response to DSBs is to facilitate the very first step of repair by promoting resection, generating the ssDNA tails required for homology search [20]. This likely involves multiple sumoylation events, as seven of the twelve proteins required for resection are sumoylated [20]. Sumoylation also regulates other steps of recombinational repair. Proteins acting after DSB resection, such as Rad52, Rad59 and RPA, are also sumoylated in response to DNA damage, and sumoylation of Rad52 in yeast and RPA in human cells have both been shown to promote recombination in specific assays [16,22,24,27]. Furthermore, defects in the SUMO E2 Ubc9 and the SUMO ligase Mms21 lead to the accumulation of joint recombination molecules, indicating that recombination has not been completed effectively or excessive recombination has been initiated [25]. This phenotype suggests that sumoylation can promote the resolution of these structures and/or channel recombination into pathways that generate fewer joint molecules. Besides HR, sumoylation also contributes to other repair processes such as nucleotide excision repair (NER) and base excision repair (BER) [21,28].

**Figure 2:**
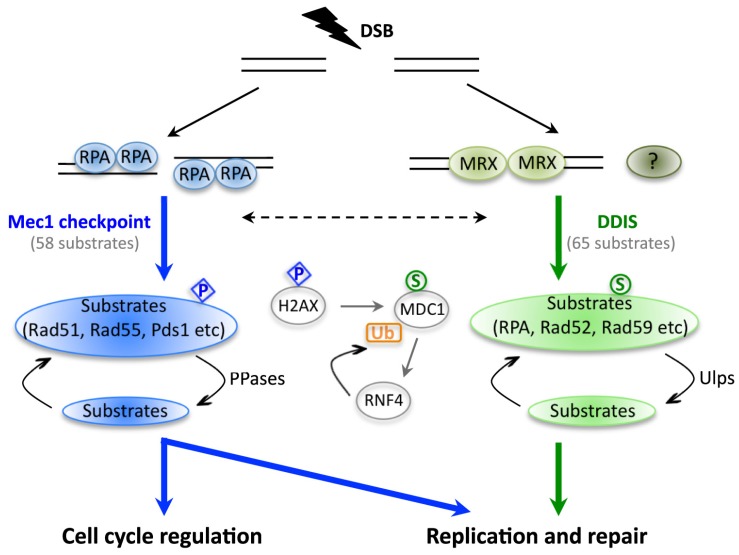
Simplified scheme showing DDIS and its possible integration within the response to DNA damage, such as DSBs. Components of the checkpoint and sumoylation responses are colored blue and green respectively. The Mec1 checkpoint is activated by long stretches of RPA-coated ssDNA, whereas MRX and possibly other factors (?) are required for DDIS. For simplicity, the checkpoint contributions of MRX and Tel1 are not depicted. The number of Mec1 checkpoint and DDIS substrates are based on systematic studies in budding yeast under several DNA damage conditions including DSBs [20,38]. P, S and Ub indicate phosphorylation, sumoylation and ubiquitination events, respectively. An example of the interplay between these modifications involving H2AX, MDC1, and RNF4 is shown as described in the text. Grey arrows specify recruitment to DSBs. Phosphatases (PPases) and desumoylating enzymes (Ulps) remove phosphate and SUMO respectively, enabling reversibility of the modification. Note that sumoylation is required for efficient generation of RPA-coated ssDNA at DSBs, thus indirectly affecting Mec1 checkpoint activation (not shown). However, DDIS is largely not required for cell cycle regulation in other DNA damage conditions, such as MMS.

However, these widespread effects of sumoylation do not tell us whether DDIS is an independent DNA damage signaling mechanism in its own right, or an outcome of other signaling pathways or repair processes. In the first scenario, DDIS responds directly to DNA damage and helps to trigger repair independently of other DDR pathways. In this case, the induction mechanism may be directly linked to damage sensing and increase the sumoylation of several proteins at once. In support of this model, many DDIS events in yeast do not rely on the known DNA damage checkpoint signaling kinases, but partly depend on upstream damage detectors such as the Mre11-Rad50-Xrs2 complex (MRX) (Figure 2; [20,24]). Currently the precise mechanism by which the damage signal is transmitted to the relevant enzymes and/or substrates to increase sumoylation is unknown. However, because MRX is required for sumoylation induction of a group of HR proteins, and deletion of its end-clipping partner Sae2 also reduces substrate sumoylation [20], ssDNA generation may contribute to the signaling process. Since deletion of the ATM homolog Tel1 has little effect on sumoylation, it is unlikely that the Tel1 recruitment function of MRX plays a key role. In the second scenario, elevated sumoylation of replication and repair proteins under damage conditions is not a signal to initiate repair, but is a consequence of increased repair that is ongoing. This possibility implies that sumoylation is a normal event that occurs as part of that protein’s repair function, and repair is upregulated independently. In this case, the induction mechanism may be indirect and vary for individual proteins. For instance, certain proteins need to be recruited to DNA in order to be modified by SUMO, suggesting that a separate recruitment signal is responsible for the increase in sumoylation. An example of this is the mammalian ATM checkpoint mediator protein MDC1, whose recruitment to damage sites via binding to phosphorylated H2AX is required for its radiation-induced sumoylation (Figure 2; [23]). Both models could explain the long-standing observation that cells defective in sumoylation are hypersensitive to DNA damage [14,15,20,25,26,29,30,31]. The timing of the modifications detected—*i.e*., sumoylation levels increasing with length of damage treatment (at least from 30 minutes to 2 hours) [22,24] and persisting for hours after the damaging agent is removed—could support either theory. It is likely that both scenarios are true, and DDIS could act as both a trigger and an effector mechanism depending on the substrates and repair processes involved.

As widespread sumoylation has previously been reported for other stress conditions, such as heat shock and oxidative stress [32,33], it is possible that increased sumoylation is a general cellular response to stress of any kind, and does not specifically respond to DNA damage. Modification events that are not ordinarily crucial could perhaps provide extra ‘backup’ in unfavorable conditions. For example, there is some evidence that increased sumoylation in the mouse brain may be protective against ischemic damage [34]. However, the nature of the protection afforded by increased general sumoylation is unclear, and yeast mutants that accumulate sumoylated species, such as *ulp2Δ* or *slx8Δ*, are not resistant to stress [27,35,36]. Also arguing against this ‘generalized stress’ theory are the examples of specificity: the fact that sumoylation targets a subset of proteins under specific conditions. For example, the human ubiquitin ligase RNF168 is sumoylated in response to IR but not UV [37]. While some NER proteins are sumoylated in response to both UV and MMS, a few are specifically modified in only one of these conditions [20,21]. It has also been suggested that increased sumoylation in stress conditions may be an indication of sick and dying cells and is not a functional and helpful response. However, sumoylation induction can also be detected in cells treated with low doses of genotoxins that would go on to recover from damage, although higher doses generally increase levels of modified protein [20,21]. Together with the defects in coping with DNA damage in sumoylation-deficient mutants, this argues that damage-induced sumoylation does occur and is functional under non-lethal damage conditions.

## 3. The Effects of Sumoylation on DNA Repair Proteins

The effect of sumoylation on each substrate is difficult to predict, given the variety of different outcomes discovered so far for the handful of substrates whose sumoylation sites have been characterized. Sumoylation site identification is at present the limiting factor for such studies, since detecting bona fide sumoylated species using current mass spectrometry techniques is challenging [39,40]. A large proportion of sumoylation sites do not conform to the consensus (ψKXD/E) motif, making educated guesswork difficult [33,41]. Compounding these issues is the variety of modification patterns that may occur on a single protein (mono- or polysumoylation at different residues) and the possibility of alternative lysines being used if the regular sites are mutated. As with ATR/ATM-induced checkpoint phosphorylation events, the effects of a single modification at a specific site may be subtle, and thus the absence of one sumoylation event may not result in an obvious damage sensitivity phenotype. Given the observation that sumoylation often targets several proteins acting in the same step of DSB repair, or multiple subunits of a complex [20,42], the effects of sumoylation may be achieved through the simultaneous modification of multiple functionally related proteins. These concerns make it challenging to dissect the roles of sumoylation of each substrate, and identify those substrates whose sumoylation contributes most to cell survival under particular conditions. 

Despite these obstacles, progress has been made in uncovering the effects of sumoylation on individual proteins. SUMO modification may alter intrinsic protein properties such as enzymatic activity, as with BRCA1 [14,15], or DNA binding ability, as with Rad52 and the BER enzyme thymine DNA glycosylase [16,28]. More examples are discussed in the accompanying review from Altmannova *et al*. [17]. Perhaps the most interesting function of sumoylation with regard to the DNA damage response concerns changes in protein-protein interactions, since repair proteins are sequentially assembled into large ‘damage foci’ following DNA damage in both yeast and mammalian cells [13,43]. Like its cousin ubiquitin, SUMO can be recognized by proteins with the cognate binding domain (SUMO Interaction Motif or SIM), allowing a modified protein to recruit cofactors. The SUMO-SIM interaction has been implicated for several functions of PCNA, the DNA replication polymerase clamp. During repair of replication blockage in budding yeast, sumoylated PCNA interacts with the DNA helicase Srs2, which dismantles Rad51 recombination filaments and thus generally disfavors recombination [44,45]. This regulation is thought to give way to other repair pathways that are less risky for the cell. Sumoylated PCNA also interacts with the alternative clamp loader subunit Elg1, thought to be involved in the unloading of sumoylated PCNA from DNA [46]. Both Srs2 and Elg1 contain SIMs in tandem with PCNA-interacting motifs that together are important for recognition of sumoylated PCNA [46,47,48]. More evidence for the importance of SUMO-SIM interactions comes from the observation that SUMO-SIM blocking peptides sensitize mammalian cells to ionizing radiation and inhibit non-homologous end joining repair of DSBs, suggesting that sumoylation-dependent interactions either have a direct impact on DSB repair or influence it indirectly [49,50]. The importance of this type of cofactor binding is also underlined by the discovery of SUMO-like domains and the corresponding SUMO-like interaction domains in other protein partners. For example, a SUMO-like domain on the deubiquitinating enzyme regulator UAF1 binds to a SIM on the Fanconi anemia protein FANCI, promoting deubiquitination of FANCI’s partner FANCD2 and subsequent interstrand crosslink repair [51]. In addition, a SUMO-like domain on the *S. pombe* protein Rad60 can bind to the SUMO conjugation enzyme Ubc9 and is involved in the removal of covalent Top1-DNA adducts [52]. 

## 4. Sumoylation Dynamics

It is simplistic to state that DNA damage-induced sumoylation must always facilitate repair, since both sumoylation and desumoylation are likely to contribute to substrate function (Figure 2). Mutations in both sumoylation and desumoylation enzymes have deleterious effects on cell growth and DNA damage sensitivity [6,35,53]. The fact that sumoylated forms are difficult to preserve in cell extracts and constitute only a small proportion of total protein at any time suggests that sumoylation is a particularly dynamic type of modification. Transient sumoylation has been suggested to have permanent effects on substrate conformation [54]; alternatively, timely sumoylation and desumoylation may trigger reversible changes in substrates in order to facilitate multi-step processes. For example, Rad52 sumoylation inhibits DNA binding and might therefore be required to assist in release of the protein from DNA, after which desumoylation would return it to a DNA binding-competent form [16]. More long-lived sumoylation may influence the localization of substrates and related proteins containing SUMO interaction domains within the nucleus; for example, PML bodies aggregate many proteins using SUMO as ‘glue’, a process that is regulated by the desumoylating enzyme SENP6 [55,56]. Unrepaired DNA breaks have also been reported to move to nuclear pores, where one or more sumoylated proteins may be recognized by SIM interactions with Slx5 [12,57]. Slx5, along with its partner Slx8, has been proposed to ubiquitinate sumoylated proteins and promote their turnover, although the identity of these putative substrates is as yet unknown.

Sumoylation and desumoylation may be controlled at the individual substrate level, or more centrally at the level of the enzyme. These methods of regulation are not mutually exclusive, and elements of both enzyme and substrate control are likely to occur locally at damage sites. Modification according to the status of the substrate occurs in the case of MDC1, which is sumoylated only when bound to phosphorylated H2AX (Figure 2; [23]). The clearest example of enzyme-level regulation is the recruitment of PIAS proteins to DSB sites [14,15], which presumably increases sumoylation of several substrates at once. However, since desumoylating activity is high in the cell, it has been suggested that the major effect of genotoxic stress may be to protect substrates from desumoylation rather than increase their sumoylation [58]. One example where control is both substrate-specific and desumoylation-dependent is the single-strand DNA binding protein RPA, whose RPA70 subunit dissociates from the SUMO protease SENP6 following camptothecin treatment, allowing more stable sumoylation [22]. Because the binding of sumoylated proteins to their SIM-containing partners can shield the SUMO moiety from desumoylating enzymes, an attractive possibility is that positive feedback could maintain or amplify the DDIS signal when initial sumoylation events are stabilized by protein-protein interactions. 

## 5. Crosstalk with other Posttranslational Modifications

Phosphorylation, as the modification responsible for the DNA damage checkpoint kinase cascade, is a prime candidate for signaling damage to sumoylation targets and/or enzymes, and could be indirectly responsible for DNA damage-induced sumoylation. However, more than one study has shown that checkpoint signaling and DDIS are separable. For example, genotoxic stress-induced sumoylation of the *NF-κB* modulator NEMO is ATM-independent [59]. In budding yeast, sumoylation of repair substrates does not require phosphorylation by the central checkpoint kinases Mec1^ATR^ or Tel1^ATM^, providing strong evidence that checkpoint and DDIS are not interdependent (Figure 2; [20]). This conclusion does not exclude the possibility of cross-talk between the two pathways, and a few examples are given below. In yeast, Mec1^ATR^ deletion in budding yeast greatly increases the sumoylation of many repair substrates at lower levels of damage [20]. The explanation for this phenomenon is still obscure, but it is possible that desumoylation could be controlled by a Mec1^ATR^-dependent phosphorylation mechanism, even though sumoylation may be induced via other means. In support of this idea, transcription of the desumoylating enzyme for NEMO, SENP2, is induced in an ATM-dependent manner [60]. Conversely, sumoylation may also affect some damage-induced phosphorylation. Mutation of sumoylation enzymes results in a modest delay in phosphorylation of the effector kinase Rad53^Chk2^ in response to MMS and a stronger delay after DSBs [20]. It was also reported that mutation of the desumoylating enzyme Ulp2 results in a slightly delayed checkpoint response and recovery, although others have found that Rad53^Chk2^ is dephosphorylated with WT kinetics [61,62]. Another study found that sumoylation of the RNA Pol II subunit Rpb1 restrains Rad53^Chk2^ phosphorylation in yeast deficient for transcription-coupled repair [63]. Finally, both Mec1 and DDIS regulate replication and repair, where more extensive cross-talk can be expected (Figure 2). It is clear that further work is needed to obtain a full picture of the interplay between the two modifications and to distinguish direct from indirect effects. 

Since the discovery that PCNA can be modified by either SUMO or ubiquitin on the same lysine residue, the interaction between these two types of modifications has been in the spotlight. As illustrated in this case, SUMO and ubiquitin modifications on the same protein are generally not competitive, though there are a few examples where the two modifications have an antagonistic relationship [64,65]. More complex crosstalk between the two systems has also emerged, notably the ubiquitination of sumoylated targets recognized by SIM-containing ubiquitin E3 ligases known as SUMO-targeted ubiquitin ligases (STUbLs). The outcome of this ubiquitination is thought to be proteasomal degradation of the sumoylated target and recycling of the modification [11,66], though examples of direct targets shown to be regulated in this way have thus far been few. Now, three labs have independently shown that the DNA damage adaptor protein MDC1 is sumoylated and is a target for the mammalian STUbL RNF4 (Figure 2; [23,67,68]). Ubiquitination and degradation of sumoylated MDC1 removes it and its binding partner 53BP1 from the damaged locus, thereby allowing recruitment of downstream recombination proteins including CtIP, RPA and Rad51 [23,67,68]. Other examples of sumoylation promoting ubiquitin ligation at DSBs include the increased catalytic activity of BRCA1 when sumoylated [14,15], and the recruitment of the ubiquitin E3 subunit BMI1 to damage sites following sumoylation by CBX4, in mammalian cells [69]. Interestingly, recruitment of CBX4 to IR damage depends on poly(ADP-ribosyl)ation, rather than phosphorylation or ubiquitination [69].

Another posttranslational modification enzyme shown to change its interaction with targets based on their sumoylation status is the deacetylase HDAC3, which preferentially binds and deacetylates sumoylated HIPK2, a p53-regulating kinase [70]. In support of an antagonistic relationship between sumoylation and acetylation, a new study from Ullmann and colleagues has shown that acetylation of SUMO itself can prevent its interaction with certain SIM-containing partners [71]. They suggest that acetylation of SUMO may be a mechanism to release SUMO-SIM binding, thereby allowing access to desumoylation enzymes. Although this additional layer of control has so far not been observed for DNA repair substrates, the authors note that the interface between SUMO and SIM in the repair enzyme thymine DNA glycosylase contains potentially acetylated residues, suggesting that examples of such combined regulation relevant to repair remain to be discovered.

## 6. Concluding Remarks

Although it is clear that many questions remain unanswered, the pace of research into the functions of posttranslational modifications in DNA repair has accelerated in recent years. Gradually, mechanistic explanations for the long-observed role of sumoylation in the DNA damage response are emerging. Though many of these studies will have to contend with extensive inter-regulation between sumoylation and other modifications as the picture becomes more complex, the recent discoveries of new sumoylation substrates involved in replication and repair should provide a good starting point to look for further molecular mechanisms that contribute to the DNA damage response.
